# Unlocking Female Fertility with a Specific Reproductive Exercise Program: Protocol of a Randomized Controlled Clinical Trail

**DOI:** 10.3390/life15010018

**Published:** 2024-12-27

**Authors:** Barbara Petra Kovács, Júlia Balog, Barbara Sebők, Márton Keszthelyi, Szabolcs Várbíró

**Affiliations:** 1 Doctoral College, Semmelweis University, 1085 Budapest, Hungary; 2 Workgroup for Science Management, Semmelweis University Doctoral College, 1085 Budapest, Hungary; sebok.barbara23@gmail.com (B.S.); keszthelyi.marton@semmelweis.hu (M.K.); varbiro.szabolcs@semmelweis.hu (S.V.); 3 Department of Metabolism, Digestion and Reproduction, Imperial College London, London W12 0NN, UK; j.balog@imperial.ac.uk; 4 Dr. Manninger Jenő Trauma Center, 1081 Budapest, Hungary; 5 Department of Obstetrics and Gynecology, Faculty of Medicine, Semmelweis University, 1082 Budapest, Hungary; 6 Department of Obstetrics and Gynecology, Faculty of Medicine, Szeged University, 6720 Szeged, Hungary

**Keywords:** infertility treatment, physiotherapy, targeted exercise, DOR, fertility improvement, FSH, AMH, randomized controlled clinical trial, combined supplements, ovarian rejuvenation

## Abstract

According to World Health Organization (WHO) data, 16% of people are affected by infertility across the globe. One underlying factor is the age-related decline of ovarian reserve (DOR), which can lead to a higher chance of infertility and has no widely accepted treatment currently. Specific supplements and moderate exercise have been shown to improve fertility; however, there is no consensus to date on the type of exercise providing the best results. Our goal is to develop a novel exercise program combined with natural supplements for the improvement of fertility. We also propose a single-centered, randomized, open-label clinical trial using our newly developed exercise in the intervention group, compared to walking and no exercise in the other groups, to investigate the benefits of this exercise program in the future. In this study, we developed a structured, novel combination of exercises focusing on the pelvic and ovarian regions, core strengthening and improvement of blood circulation in this region. The 70 min full body “reproductive gymnastics”, includes strengthening, stretching, and relaxation exercises combined with yoga-inspired moves and diaphragmatic breathing with meditation elements to activate the parasympathetic pathway and stress relief. We believe we can improve fertility through the combination of natural supplements and our targeted, moderate physiotherapy program in women with DOR.

## 1. Introduction

Infertility is a growing concern worldwide, marked by the inability to achieve clinical pregnancy after a year of regular, unprotected intercourse [1]. Its global prevalence is significantly increasing, concurrently with the rising trend of delayed parenthood, and it affects an estimated 186 million people [2,3]. With increased maternal age, patients have to confront the decline in age-related ovarian reserve (DOR), which is well defined by the current determination of Anti-Müllerian Hormone (AMH), Antral Follicle Count (AFC), and basal FSH levels [4]. According to the American Society for Reproductive Medicine, the use of AMH and AFC as screening tests is most suitable for detecting poor ovarian response [5]. Since no single measure of ovarian reserve has 100% specificity in diagnosing DOR, a combination of biochemical and transvaginal ultrasound measurements (including FSH, LH, E2, AMH, and AFC) is utilized in this study to evaluate changes in ovarian reserve. Diminished ovarian reserve (DOR) is characterized by poor fertility indicators, even with the application of assisted reproductive techniques (ARTs), posing a significant challenge in reproductive medicine [6].

It has been shown that supplements have a high impact on the restoration of fertility. Myo-inositol has been demonstrated to improve ovarian function and increase the quality of oocytes, particularly in women with polycystic ovary syndrome (PCOS), leading to higher pregnancy rates [7,8]. In particular, several clinical trials demonstrate that its administration can have therapeutic effects in infertile women, and that it can also be useful as a preventive treatment during pregnancy [9].

Folic acid is essential for DNA synthesis and repair, and its supplementation is crucial for proper fetal development and reducing the risk of neural tube defects [10]. Melatonin not only regulates sleep but also acts as a potent antioxidant, protecting oocytes from oxidative damage and improving their quality [11,12,13]. Vitamins C and E, both powerful antioxidants, have been shown to reduce oxidative stress in the reproductive organs, thereby enhancing fertility, not only in woman [14,15]. Vitamin D3 plays a critical role in the modulation of the immune system and the regulation of reproductive processes, with deficiencies linked to adverse fertility outcomes [16]. Coenzyme Q10 is vital for mitochondrial function and energy production in oocytes, with studies indicating that its supplementation improves ovarian response and embryo quality [17,18]. Collectively, these supplements contribute to a comprehensive approach to restoring and enhancing fertility, addressing both physiological and biochemical factors involved in reproductive health.

The World Health Organization (WHO), and the American College of Obstetricians and Gynecologists (ACOG) suggests that women planning pregnancy should engage in at least 150 min of moderate physical activity per week, to reduce reproductive risks [19,20]. Not only does engaging in regular exercise improve fertility outcomes, but the specific type of physical activity plays a major role in enhancing reproductive health.

The coordinated, optimal functioning of the muscles involved in pelvic-lumbar (core) stabilization (diaphragm, pelvic floor muscles, transversus abdominis muscle (TVA), multifidus muscles) is of paramount importance for spinal protection, efficient breathing and movement, injury prevention, force transmission, optimal function and support of the pelvic floor and pelvic organs (ovaries, uterus), and proper regulation of sexual function. Pelvic-lumbar (core) stabilization is developed by strengthening the deep stabilization system of the spine, in coordination with the correct breathing pattern [21]. The proper muscle function of the TVA is of paramount importance for pelvic and lumbar stability, the restoration of physiological breathing patterns, and the efficient functioning of the pelvic floor muscles that work in co-contraction with it. Rehabilitation of the pelvic region and pelvic floor is essential to improving fertility. Exercises that improve pelvic-lumbar (core) stabilization have been shown to improve the function of the pelvic floor muscles, which play a crucial role in supporting the reproductive organs and improving blood supply to the ovaries and uterus [22]. Several authors agree that through breathing exercises, the complex trunk stabilization system can be directly affected [23,24,25]. Normal breathing mechanics play a key role in posture, optimal pelvic floor muscle function, and spinal stabilization.

Prolonged mental and physical stress, postural and lifestyle habits, and improper breathing patterns all contribute to diaphragmatic dysfunction. These factors also stimulate the sympathetic nervous system and stimulate the secretion of the stress hormone (cortisol), which also has a negative impact on fertility [26]. Additionally, diaphragmatic breathing has been shown to reduce stress, which is crucial for enhancing fertility, as chronic stress can disrupt hormone balance and menstrual cycles [27]. Yoga and diaphragmatic breathing, in particular, have demonstrated stress-reducing benefits, activating the parasympathetic nervous system and promoting a state of relaxation conducive to reproductive health [28,29].

These professional aspects should be combined in a targeted and comprehensive multidimensional exercise program.

Despite these promising findings, further research is needed to confirm these effects due to the limited number and sample size of previous studies and the current ambiguity in exercise recommendations for improving fertility outcomes.

In this study, we present a novel and innovative therapeutic exercise protocol specifically designed for pelvic region rehabilitation and ovarian rejuvenation. The therapeutic exercises focus on improving fertility, rehabilitation of the pelvic region (including re-education of the pelvic floor muscles), teaching correct posture, improving the pelvic-lumbar core stabilization system, restoring optimal muscular function of the diaphragm, and activating the parasympathetic nervous system through learning stress-relieving breathing and relaxation. These exercises are expected to enhance pelvic circulation and improve muscle function, which are crucial for reproductive health, especially in patients with diminished ovarian reserve (DOR).

The primary objective of this study is to assess the ovarian rejuvenating effect of this specific exercise program before IVF/ICSI cycles or spontaneous pregnancy, confirming the reproductive efficacy of this regimen. We also aim to improve stimulation and oocyte quality in patients who meet the inclusion criteria, partly by improving pelvic and ovarian circulation (ovarian rejuvenation exercise, the subject of this study) and partly by enhancing cumulus corona radiata and granulosa cell function (per os rejuvenation therapy).

It is expected that our results will show that AMH will be increased, basal FSH levels will be decreased, cycle length will be optimized (getting closer to 28–30 days), fertility rates and stimulation will be improved, and spontaneous pregnancy will be detected at the end of the process. If this investigation confirms the efficacy of the specific exercise program in DOR patients, this could make a useful contribution to fertility treatment in this poorly manageable patient population.

## 2. Experimental Design

This protocol was written in accordance with the Standard Protocol Items: Recommendations for Interventional Trials (SPIRIT) reporting template [30]. This was a single-center, randomized (1:1:1), open-label clinical trial, with three treatment groups: per os therapy (Group A), per os therapy and walking (Group B), per os therapy and special exercise (Group C). The purpose of randomization is to eliminate selection bias. The study protocol was approved and registered by the Human Reproduction Committee of Hungarian Medical Research Council (25489-8/2021/EÜIG).

### 2.1. Study Setting

This study will involve women aged 20–42 diagnosed with diminished ovarian reserve (DOR) who meet the following criteria: (1) AMH levels < 1.1 ng/mL at the beginning of the cycle, (2) BMI between 18.5 and 30 kg/m^2^, (3) regular and normal menstruation, and (4) specified hormone levels (TSH < 2.5 mIU/mL, vitamin D3 > 75 nmol/L, Prolactin < 24 ng/mL). We plan to enroll 120 patients between January 2025 and December 2025.

### 2.2. Eligibility Criteria

#### 2.2.1. Inclusion Criteria

Participants who meet the following inclusion criteria will be included: (1) female of reproductive age 20–42 years; (2) BMI: 18.5–30 kg/m^2^; (3) regular menstruation; (4) anti-Müllerian hormone (AMH) <  1.1 ng/mL; (5) hormone levels: TSH < 2.5 mIU/mL, vitamin D3: >75 nmol/L, Prolactin: <24 ng/mL; (6) understanding the study design, risks, and benefits, and providing informed consent; and (7) the ability to comply with the study protocol.

#### 2.2.2. Exclusion Criteria

Participants meeting any of the following criteria will be excluded: (1) age > 42 years old; (2) antral follicle count (AFC) <  3 measured on day 2 of the cycle; (3) multiple unsuccessful stimulation cycles ending with cancellation; (4) allergy to medications used for ovarian rejuvenation; (5) three or more ovarian surgeries result in significant ovarian reserve depletion (iatrogenic POI, iatrogenic DOR); (6) secondary amenorrhea; (7) uterine developmental abnormalities; and (8) inability to engage in physical activity.

### 2.3. Objectives

The primary objective of this study is to assess the efficacy of specific exercise routine on ovarian function compared with a standard of care and low intensity walking in patients with diminished ovarian reserve, by optimizing hormone levels for better stimulation possibilities.

#### 2.3.1. Primary Outcome

The primary outcome is the change in FSH and AMH levels compared with the levels before. Spontaneous pregnancy during the study period will be recorded as a primary outcome measure.

#### 2.3.2. Secondary Outcomes

The secondary outcomes include: optimization of E2 and LH levels; optimization of menstrual cycle length; change in BMI level; improvement in quality of life using the FSFI questionnaire; and comparing the effectiveness between the 3 study groups.

### 2.4. Sample Size

A parallel, 3-group design will be used. We determined the sample size using G*Power 3.1.9.7 software. We employed one-way analysis of variance (ANOVA) due to consideration of three different therapies. The effect size was estimated at 0.4. We set alpha to 0.05 and power to 0.95. Consequently, the software provided an estimated total sample size of 102, resulting in 34 cases per group. To account for a potential dropout rate of 15%, we increased the sample size to 40 participants per group. Therefore, the total sample size needed for the study is 120 participants [31].

### 2.5. Statistical Methods

Descriptively, we intend to calculate the mean, standard deviation, minimum, maximum, and range of data, while the occurrence of spontaneous pregnancy will be presented as a percentage. For the evaluation of results, after conducting the Shapiro–Wilk normality test, paired T-tests or Wilcoxon tests will be used to compare baseline and three-month laboratory results within each group, and one-way analysis of variance (ANOVA) or Kruskal–Wallis’s test, along with Tukey’s post hoc test, will be used for comparison among the three groups. Chi-square tests or Fisher’s exact tests are planned to be applied for comparing the numbers or percentages between groups.

### 2.6. Recruitment

Patients meeting the eligibility criteria will be recruited from the Assisted Reproduction Center of Semmelweis University, Hungary. Detailed information about the study will be provided verbally and in writing by the examining physician. Research subjects are informed that in this clinical trial we intend to compare three groups of infertile women. The group receiving only per os complementary therapy, the group receiving per os complementary therapy and walking, and the group receiving per os complementary therapy and a specific exercise program will be tested. We will also inform prospective participants that participation in the research is voluntary and that the anonymized data obtained will be summarized and subjected to statistical analysis. Participation in the study will take approximately 12 weeks.

### 2.7. Implementation

The principal investigator or a designated sub-investigator will input the required data into a web-based allocation system for eligible patients. Subsequently, the system will allocate participants to the three investigated groups. The required data include personal data, demographic data, medical and family history, body composition assessment (weight, height, BMI), menstrual cycle (length and days of menstruation), hormone levels (AMH, FSH, LH, E2, PRL, TSH, vitamin 25-OH-D3), progesterone levels, and the FSFI questionnaire, as shown in Table 1.

## 3. Materials and Equipment

The per os therapy consists of the following supplementation and amounts: daily 3 mg of melatonin (Pharma Nord, Vejle, Denmark) before bedtime in addition to daily intake of 1000 mg of vitamin C (slow release), 400 mg of vitamin E, 2500 IU/day of vitamin D3, and 200 mg of Coenzyme Q10 (Pharma Nord, Vejle, Denmark); and twice daily a preparation containing 2000 mg of myo-inositol, 50 mg of alpha-lactalbumin, and 200 μg of folic acid (Inofolic premium, Exeltis Hungary, Budapest, Hungary). For the rejuvenation exercise, there is no mandatory equipment, although an exercise mat and a pillow or ball are recommended.

## 4. Detailed Procedures

### 4.1. Intervention

The standard dietary supplement/vitamin oral therapy for all three groups includes the intake of a preparation containing myo-inositol + folic acid orally twice a day. Participants in the study are asked to take 3 mg of melatonin before bedtime in addition to daily intake of 1000 mg of vitamin C (slow release), 400 mg of vitamin E, 2500 IU/day of vitamin D3, and 200 mg of Coenzyme Q10.

Combined with the supplement therapy, patients in Arm B will be asked to perform low intensity walking 3 times per week for 1 h each session.

For Arm C (per os therapy and special exercise), our special 70 min moderate-intensity exercise program is available 3 times a week for at least 3 months. This therapeutic exercise program is designed to combine several professional aspects in all exercises including postural correction, pelvic-lumbar stabilization, rehabilitation of the pelvic region, re-education of the pelvic floor muscles, restoration of optimal diaphragm function, dynamic mobilization and stretching exercises, use of the relaxation and therapeutic effects of costo-diaphragmatic breathing, optimization of joint range of motion, and development of balance and coordination. There is a large emphasis on the combination of exercises with relaxation-type exercises of costo-diaphragmatic breathing. The constant attention on breathing helps the patients relax, activates the parasympathetic nervous system, and ensures the strengthening of multiple muscle groups by contraction and relaxation.

The therapeutic movement program begins with a 15 min warm-up focused on teaching active elongation and correct posture, diaphragmatic breathing, hip joint–shoulder joint–thoracic spine mobilization exercises and activating the gluteal group to prepare the pelvic region and the whole body for exercise. The warm-up is followed by a 15 min active core strengthening session, designed to develop the pelvic-lumbar stabilization system and optimize hip joint range of motion, in coordination with proper breathing technique. Proper mobility of the lumbar–pelvic–hip complex and optimal functioning of the core muscles are also necessary for optimal functioning of the pelvic organs (ovaries, uterus). In this session, exercises use coordinated activation of the TVA and pelvic floor muscles along with breathing. The third 15 in section focuses on costo-diaphragmatic breathing in various yoga postures to activate the parasympathetic nervous system, promoting relaxation and stress relief and improving fertility. The other aim of this phase is to restore muscle balance around the pelvis through stretching exercises and to relax the PF through relaxation breathing techniques. The next 15 min session before the cool-down presents the therapeutic objectives of the previous sessions in a complex way, including postural correction, dynamic stretching, and core stabilization exercises, together with correct breathing techniques. Our goal is to compensate for mobility problems due to a sedentary lifestyle, relieve pelvic floor muscles, improve pelvic organ circulation, and correct fertility problems due to restricted hip movements. The final 10 min session is an active pelvic mobilization through dance movements in a vertical position. The aim of this phase is to optimize the movements of the hypomobile lumbar–pelvic–hip complex (LPHC). This is achieved by hip rotations, pelvic tilts, and a combination of these movements. If there is not adequate range of motion in the lumbar spine–pelvic–hip joint, sexual function is significantly affected and limited, and this also has a negative impact on fertility. The stress-relieving effects of music and dance are used to release female energy and mobilize the pelvic girdle.

The workout can be tailored to specific needs by offering multiple levels and incorporating weights. All the exercises will be conducted by a women’s reproductive health physiotherapist.

### 4.2. Randomization

**Figure 1:**
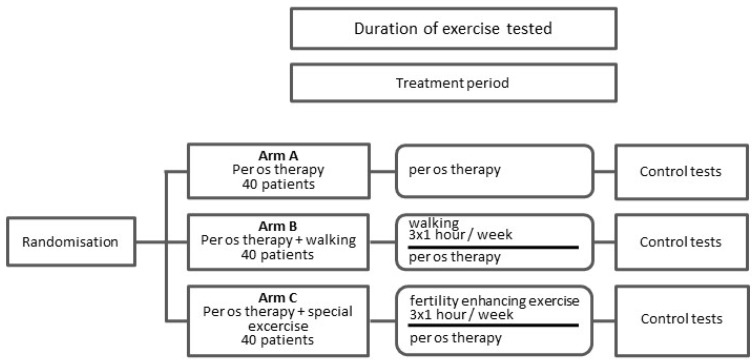
Trial design.

Patients will be randomly assigned to Arm A (per os therapy), Arm B (per os therapy and walking), or Arm C (per os therapy and special exercise) in a 1:1:1 ratio by an internet-based distant third-party statistician blinded to the study and participant details. The recruiting physician will be trained and provided with detailed instructions on the recruitment protocol. The objective of randomization is to eliminate selection bias. The trial design is summarized in Figure 1.

### 4.3. Treatments of Adverse Events

Local investigators will handle any adverse events in accordance with current good clinical practice guidelines. Each adverse event will be documented in a case report form, detailing its nature, onset and resolution times, severity, treatment, and outcome. Follow-up examinations will be conducted as needed to ensure patient safety.

Should the monitoring physician observe any harm to a participant or signs of ineffectiveness during the trial, the participant will be removed from the study.

### 4.4. Criteria for Discontinuation of Trial Treatment

The criteria for the discontinuation of a trial are as follows:

(1) The participant refuses further participation or withdraws consent; (2) termination of the entire study; (3) occurrence of a spontaneous pregnancy; (4) failure to adhere to the study rules; (5) attendance of fewer than 75% of the exercise sessions by the participant in the intervention group; (6) occurrence of any other intervention or surgery during the study period that may alter the study outcome; (7) undesirable events related to the treatment, such as acute pain, musculoskeletal complaints, or acute mental or physical trauma preventing continuation of the study; (8) presence of a newly diagnosed malignancy; (9) any situation where oral therapy or physiotherapy is deemed unfit to continue according to the decision of the physician and physiotherapist.

### 4.5. Discontinuation of the Study

The study will be terminated early if the Institutional Review Board (IRB) identifies any of the following: serious adverse drug effects; a newly diagnosed malignancy; or participants encountering unexpected, significant, or unacceptable risks (such as death).

### 4.6. Baseline Assessments

Baseline assessments will adhere to the trial’s standard operating procedure (SOP). During the first visit, between days 2 and 4 of the cycle, a laboratory examination will be conducted, including hormone levels of AMH, FSH, LH, E2, PRL, TSH, 25-OH-D3 vitamin, and progesterone. Baseline progesterone will be measured between days 21 and 22 of the same cycle. The FSFI questionnaire will also commence during this phase.

### 4.7. Efficacy Assessments

The efficacy of the trial treatment will be evaluated using a comprehensive set of parameters measured at the 3-month follow-up. Participants’ weight, height, and BMI will be recorded. This will help in assessing any changes in body composition resulting from the intervention. The regularity and characteristics of participants’ menstrual cycles will be monitored from the start to the end of the study. This continuous observation aims to identify any improvements or alterations in menstrual health. Hormonal evaluations, including levels of AMH, FSH, LH, E2, PRL, TSH, and 25-OH-D3 vitamin, will be conducted at the conclusion of the study as well. As a primary outcome, the change in FSH and AMH levels will be administered. The measurement of progesterone will help evaluate the sufficiency of the luteal phase, which is critical for reproductive health. These measures will provide insights into the hormonal balance and ovarian function of the participants. The FSFI questionnaire will be administered to assess changes in sexual function. This self-reported measure will provide valuable data on the impact of the intervention on participants’ sexual health. The occurrence of spontaneous pregnancy during the study period will be recorded as a primary outcome measure. This will directly indicate the effectiveness of the intervention in improving fertility. These comprehensive assessments will collectively provide a thorough evaluation of the intervention’s efficacy.

### 4.8. Data Monitoring Committee

An oversight committee comprising clinical trial experts, including a biostatistician, will be established. The oversight committee will analyze unblinded outcome data to assess safety and efficacy, determining whether there are indications of unsafe treatments warranting the discontinuation of the trial. Additionally, the committee will alert the Trial Steering Committee to any instances of unethical treatment or serious adverse events.

To manage the data for this study, the IBM Clinical Development Management System (IBM Corporation, Somers, NY, USA), utilizing an Electronic Data Capture (EDC) system, will be employed.

## 5. Expected Results

This clinical trial aims to explore whether our therapeutic exercise program designed to rejuvenate the ovaries and rehabilitate the pelvic region is effective in improving ovarian reserve in women with DOR. The exercise aims to show the effects of increased pelvic region circulation on ovarian reserve. We are not aware of any other studies aiming to discover the effects of pelvic-specific physical therapy on the fertility of women with DOR. Our study is the first to investigate the multifaceted impact of specially combined physical activity compared with walking and no physical activity at all on female fertility in patients with infertility diagnosed with diminished ovarian reserve (DOR). Improving assisted reproductive outcomes has become a critical issue for both infertile couples and clinicians. Considering the strong desire of patients and the urgent need for treatment, per os rejuvenation therapy combined with walking and per os rejuvenation therapy “without exercise” were selected as a control method rather than placebo. Targeted exercise of the pelvic region and the relaxation and therapeutic effects of costo-diaphragmatic breathing may be an important factor besides lifestyle changes and nutritional factors, which are among the most valuable and promising interventions in preserving and restoring human health and fertility, representing an area of medical research that remains to be fully explored [32].

Data on the association between physical activity and female fertility are subject to debates. Several studies have previously investigated the therapeutic effects of exercise on fertility, but no clear consensus has emerged. In many studies, researchers have reported mixed results, which is probably one of the reasons why the factors affecting fertility are multifactorial. Several studies show that regular, moderate physical activity can help achieve optimal hormonal balance and regular ovulation, thereby improving fertility [33,34]. Some clinical and experimental studies have also revealed that regular moderate intensity exercise is beneficial for maintaining and improving ovarian reserve and positively influences the ovarian reserve profile [35,36]. There is also evidence available that physical activity combined with other psychosocial and metabolic stressors can trigger a stress response, inhibiting ovulation and the production of key hormones for conception [34]. Frequent high-intensity physical activity (PA) increases subfertility and infertility, especially ovulatory infertility [37]. Athletes have been observed to have a higher incidence of reproductive dysfunction and ovulatory infertility than non-athletes [38,39]. However, there are also studies showing the opposite effect, i.e., that vigorous PA may be associated with improved fertility, although this has only been shown in women with low physical activity, overweight, and obesity [33,40]. There is also no consensus among researchers on the effect of physical activity before ART on pregnancy outcomes [41,42,43]. In a systematic review of 34 studies published in 2023, researchers concluded that there was insufficient evidence to determine the relationship between physical activity and male and female fertility [44]. This review also examined the association between daily walking duration and female fertility. Conflicting results were found when examining this relationship.

The recommendation of the World Health Organization (WHO) and the American College of Obstetricians and Gynecologists (ACOG) that women planning a pregnancy should do at least 150 min of moderate physical activity per week to reduce reproductive risk [19] is in accordance with our study recommendations. Despite a large number of studies on the curative relationship between physical activity and fertility, in fact, we still know very little about the optimal duration, intensity, and form of PA in reducing infertility risk. The goal of our study is to optimize and rigorously test the effect of a reproductive, targeted exercise on ovarian function in a controlled environment, and possibly include specific exercises in the treatment options for DOR and infertility.

In conclusion, our study addresses the type of physical activity in enhancing various aspects of female fertility, including body composition, hormonal balance, menstrual regularity, sexual function, and spontaneous pregnancy rates. These findings advocate for the integration of regular therapeutic exercise into fertility treatment plans to optimize reproductive health outcomes. Our preliminary three volunteer patients had a promising change in their FSH and AMH levels after 3 months of exercise, further supporting the possible success of the study. Our study results may provide high-quality evidence for evaluating the effectiveness of reproductive physiotherapy in the treatment of DOR patients and in the evaluation of outcomes following IVF-ET. This study will contribute to providing a therapeutic option for DOR patients.

## Figures and Tables

**Table 1:** Study Assessments and Procedures.

	Screening	Baseline (2–4. Day of Menstrual Cycle)	Baseline (21–22. Day of the Same Cycle)	3 Months (2–4. Day of Cycle)	3 Months (21–22. Day of the Same Cycle)
Assessment					
Investigator meetings	✓				
Informed consent	✓				
Demographics, medical, and family history	✓				
Body composition assessment (Weigh, Height, BMI)	✓			✓	
Menstrual cycle	✓			✓	
Hormone levels (AMH, FSH, LH, E2, PRL, TSH, 25-OH-D3 vitamin)		✓		✓	
Progesteron		✓	✓		✓
FSFI questionnaire		✓		✓	
Spontaneous pregnancy				✓	

## Data Availability

Data are available at the request from the authors.
